# Supplementary material for: Single Cell Transcriptomic and Chromatin Profiles Suggest Layer Vb Is the Only Layer With Shared Excitatory Cell Types in the Medial and Lateral Entorhinal Cortex
Source: Front Neural Circuits. 2022 Jan 26;15:806154. doi: 10.3389/fncir.2021.806154 (PMC8826650; doi:10.3389/fncir.2021.806154)
Supplement: Supplementary file 14 [file Data_Sheet_1.ZIP › MEC_Sup_vs_LEC_Sup_Motifs/homerResults/motif5.similar.html]

motif5

## Information for motif5

G
C
A
T
C
G
T
A
A
G
T
C
G
T
C
A
A
G
C
T
T
G
C
A
A
G
C
T
C
G
T
A
A
G
C
T
T
C
G
A
  
Reverse Opposite:  

A
G
C
T
T
C
G
A
C
G
A
T
T
C
G
A
A
C
G
T
T
C
G
A
C
A
G
T
T
C
A
G
G
C
A
T
C
G
T
A
  

|  |  |
| --- | --- |
| p-value: | 1e-75 |
| log p-value: | -1.740e+02 |
| Information Content per bp: | 1.498 |
| Number of Target Sequences with motif | 4475.0 |
| Percentage of Target Sequences with motif | 21.23% |
| Number of Background Sequences with motif | 1997.2 |
| Percentage of Background Sequences with motif | 16.36% |
| Average Position of motif in Targets | 400.6 +/- 261.5bp |
| Average Position of motif in Background | 361.1 +/- 232.4bp |
| Strand Bias (log2 ratio + to - strand density) | -0.0 |
| Multiplicity (# of sites on avg that occur together) | 1.29 |
| Motif File: | file (matrix) reverse opposite |

### Similar de novo motifs found

|  |  |  |  |  |  |  |  |
| --- | --- | --- | --- | --- | --- | --- | --- |
| Rank | Match Score | Redundant Motif | P-value | log P-value | % of Targets | % of Background | Motif file |
| 1 | 0.635 | C G T A G C A T C G T A C G A T C T A G G A T C G C T A G C A T C T G A G A C T C G T A G A C T | 1e-69 | -161.057912 | 20.09% | 15.51% | motif file (matrix) |
| 2 | 0.615 | A G C T C T G A A C G T C T A G A G C T C G T A G T C A A T C G | 1e-38 | -88.886969 | 12.88% | 10.07% | motif file (matrix) |
